# The soft mechanical signature of glial scars in the central nervous system

**DOI:** 10.1038/ncomms14787

**Published:** 2017-03-20

**Authors:** Emad Moeendarbary, Isabell P. Weber, Graham K. Sheridan, David E. Koser, Sara Soleman, Barbara Haenzi, Elizabeth J. Bradbury, James Fawcett, Kristian Franze

**Affiliations:** 1 Department of Physiology, Development and Neuroscience, University of Cambridge, Downing Street, Cambridge CB2 3DY, UK; 2 Department of Biological Engineering, Massachusetts Institute of Technology, 77 Massachusetts Ave 56, Cambridge, Massachusetts 02139, USA; 3 Department of Mechanical Engineering, University College London, London WC1E 7JE, UK; 4 School of Pharmacy and Biomolecular Sciences, University of Brighton, Lewes Road, Brighton BN2 4GJ, UK; 5 John van Geest Centre for Brain Repair, University of Cambridge, Robinson Way, Cambridge CB2 0PY, UK; 6 Wolfson Centre for Age-Related Diseases, King's College London, Guy's Campus, London SE1 1UL, UK

## Abstract

Injury to the central nervous system (CNS) alters the molecular and cellular composition of neural tissue and leads to glial scarring, which inhibits the regrowth of damaged axons. Mammalian glial scars supposedly form a chemical and mechanical barrier to neuronal regeneration. While tremendous effort has been devoted to identifying molecular characteristics of the scar, very little is known about its mechanical properties. Here we characterize spatiotemporal changes of the elastic stiffness of the injured rat neocortex and spinal cord at 1.5 and three weeks post-injury using atomic force microscopy. In contrast to scars in other mammalian tissues, CNS tissue significantly softens after injury. Expression levels of glial intermediate filaments (GFAP, vimentin) and extracellular matrix components (laminin, collagen IV) correlate with tissue softening. As tissue stiffness is a regulator of neuronal growth, our results may help to understand why mammalian neurons do not regenerate after injury.

## Abstract (web summary)

Glial scars are thought to provide a biochemical and mechanical barrier to neuronal regeneration post-injury, but the mechanical properties of the scars have not been studied in detail. Here the authors perform atomic force microscopy measurements of glial scars from the injured rat cortex and spinal cord, and find that brain tissue softens in response to the injury.

Traumatic injury to the body is usually followed by blood clotting, inflammation, cell proliferation, and tissue remodelling. Physiological repair mechanisms, activated after damage, strike a fine balance between wound closure/healing (a relatively fast event that promotes survival of the whole animal) and regeneration of new cells/tissue (a process that takes longer but results in functional recovery of the damaged site). In adult higher vertebrates, which cannot regenerate most of their organs, injuries often lead to fibrosis, and wounds result in some degree of scarring. Scars are typically rich in strongly cross-linked collagen-1, and as the amount of collagen-1 determines the stiffness of a tissue^1^, scars are normally considerably stiffer than healthy tissue in a variety of organs^2^,^3^, including skin^4^,^5^, heart^6^,^7^,^8^, blood vessel^9^ and liver^10^.

In the central nervous system (CNS), glial cells are responsible for the local immune response and wound healing processes. Here, injuries similarly lead to the formation of scars, commonly referred to as ‘glial scars'. However, these ‘glial scars' are comprised not only of glial cells, such as astrocytes and NG2-glia, but also of non-neural cells, such as pericytes and meningeal cells^11^,^12^, as well as of extracellular matrix (ECM). Glial scars are required to seal the site of injury, protect the damaged neural tissue, prevent an overwhelming inflammatory response, and to re-establish the blood–brain barrier^13^,^14^,^15^. On the other hand, it is also the scar which may prevent injured axons from regenerating past the lesion^14^,^15^. Glial scars impair axonal outgrowth, which causes aberrant function or death of neurons, leading to devastating consequences such as permanent paralysis of patients after spinal cord injuries.

Glial scars are thought to provide not only a biochemical but also a mechanical barrier to neuronal regeneration^15^,^16^,^17^. In addition to repressive ECM molecules and other signalling molecules produced by glial scars, such as chondroitin sulphate proteoglycans (CSPGs)^18^, a dense meshwork of cells and ECM allegedly constitute a ‘stiff' obstacle which neurons cannot penetrate^17^.

The stiffness of the environment is indeed an important determinant of neuronal and glial cell growth and function. *In vitro*, spinal cord neurons show increased branching on softer substrates^19^. While hippocampal neurite length is independent of substrate stiffness, neurites of dorsal root ganglion cells grow longer on stiffer substrates^20^. In co-culture, softer substrates select for growth of cortical neurons over that of astrocytes, and, vice versa, on stiffer substrates astrocyte growth is enhanced^21^. In other studies, spinal cord astrocytes adapted their morphology to their mechanical environment^22^, while neurons had fewer dendrites but longer axons on softer substrates^23^. Microglial cells, the primary immune cells of the CNS, are mechanosensitive as well, and adapt their morphology and migration behaviour to the stiffness of their environment^24^,^25^. *In vivo*, stiff implants trigger significantly enhanced levels of glial cell activation and subsequent inflammation (that is, gliosis) compared to softer implants^25^,^26^. Furthermore, retinal ganglion cell axon growth and guidance in the developing *Xenopus* brain are controlled by local gradients in tissue stiffness^27^. The body of evidence for an involvement of mechanical signals in controlling neuronal growth and possibly regeneration, as well as glial cell activation, is continuously growing^28^,^29^.

Any changes in the mechanical properties of CNS tissue post-injury may thus have important consequences for neuronal regrowth, and knowledge about such changes might inform new approaches aimed at facilitating neuronal regeneration. However, to date the mechanical properties of glial scars remain enigmatic. While it is commonly assumed that glial scars are stiff like other scars, collagen-1, which scales with tissue stiffness^1^, is limited in the CNS to some basal laminas, and it is absent from glial scars^30^. Using atomic force microscopy (AFM) indentation experiments, here we show that, in contrast to all other known scars, glial scars in both the rat cortex and spinal cord are softer than healthy CNS tissue. Furthermore, we illuminate changes in ECM and glial cell protein expression that accompany changes in tissue mechanics and identify vimentin, GFAP, collagen IV, and laminin as possible key players in the softening of the scar tissue.

## Results

### Mechanical characterization of rat brain tissue

**Figure 1:**
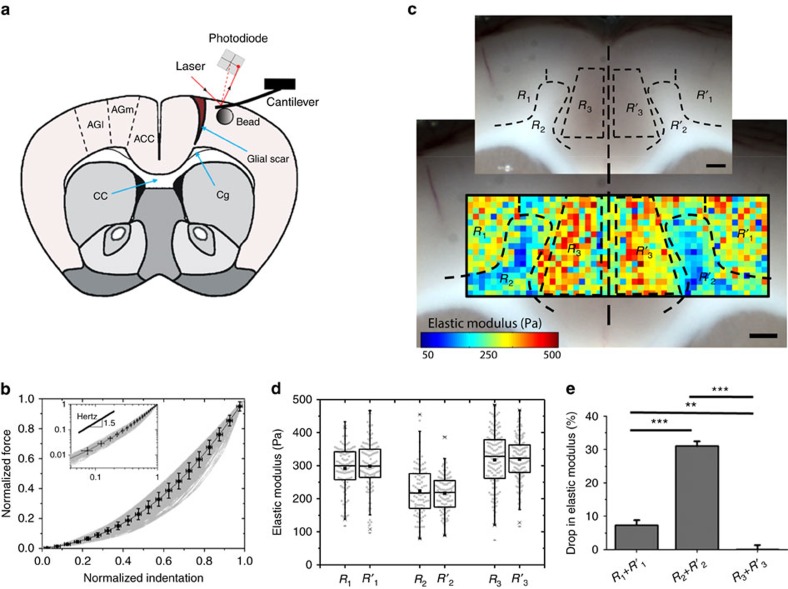
Mechanical properties of the intact cortex. (**a**) Schematic showing the location of the stab injury in the rat brain and the areas of the cortex where elasticity measurements were recorded by AFM. CC indicates corpus callosum, Cg cingulum, AGm medial agranular, AGI lateral agranular and ACC anterior cingulate regions of the cortex. An AFM cantilever indents the brain tissue; the applied force is translated to a bending of the cantilever that is readable through changes in the laser light path and detected by a photodiode. (**b**) The force–indentation curves obtained by AFM micro-indentation tests followed a spherical Hertzian contact model. For each force–indention curve, the indentation depth and force were divided by their respective maximum values. The black line passing the averaged force values serves as a guide to the eye; error bars are s.d. The inset shows the normalized force–indentation curves on a log–log scale; the curves follow a slope of ∼1.5, consistent with the slope of the Hertz contact model. (**c**) Spatial mapping of the elastic modulus of a healthy rat brain cortex. The elastic modulus at each pixel is measured by AFM indentation and is represented as a rainbow-coloured palette map with blue denoting softer and red corresponding to stiffer regions. The elasticity map and the brightfield image of the tissue are overlaid. Scale bars, 500 μm. (**d**,**e**) The elasticity map is symmetric around the midline; three regions (that is, R_1_, R_2_ and R_3_) with significantly different mechanical properties were identified in each brain hemisphere. The AGm that contains larger amounts of myelinated axons (that is, R_2_ and R′_2_) was significantly softer than the AGl (R_1_ and R′_1_) and the ACC (R_3_ and R′_3_). (**e**) The average percentage drop in elastic modulus was calculated considering the combined average elasticity of regions R_3_ and R′_3_ as the baseline. Error bars are s.e.m., **for *P*=0.003 and ***for *P*<0.001, Tukey–Kramer *post hoc* test.

To characterize the mechanical properties of the rat neocortex, we first performed micro-indentation AFM experiments on uninjured coronal brain slices perfused with artificial cerebrospinal fluid (aCSF) (Fig. 1a). Hertzian contact theory was found to be a good model to fit force–indentation curves (Fig. 1b), thus allowing us to calculate local apparent elastic moduli. Elastic modulus values, which are a measure of the tissue's elastic stiffness, ranged from 50 to 500 Pa for uninjured cortical tissue with a median value of 285 Pa (combined averaged results from 1,730 measurements of two brains with no significant difference between their stiffness) (Fig. 1c,d), confirming the relatively soft nature of the adult rat brain^28^.

Next, the spatial heterogeneity of the elastic properties of the tissue was evaluated by performing raster indentation scanning across brain slices. The rectangular elasticity maps were heterogeneous but symmetric with respect to the brain midline (Fig. 1c), indicating that tissue stiffness is tightly controlled and that the tissue retained stable mechanical properties throughout the measurements. Three mechanically distinct regions were identified on the AFM maps whose average elastic moduli appeared to be linked to the proportion of axonal tracts in the area. The medial agranular cortex (AGm) region, containing myelinated fibres of the cingulum (R_2_ and R′_2_ in Fig. 1c), was the softest region with an apparent elastic modulus of 219±65 Pa (mean±s.d., Fig. 1d, *P*<0.001, Tukey–Kramer *post hoc* test, *n*≥90 measurement points in each region, Supplementary Table 1). The lateral agranular cortex (AGl; R_1_ and R′_1_ in Fig. 1c) had an intermediate stiffness of 295±72 Pa (mean±s.d., Fig. 1d), while the anterior cingulate cortex (ACC; R_3_ and R′_3_ in Fig. 1c) nearer to the brain midline exhibited the largest elastic modulus of 318±75 Pa (mean±s.d., Fig. 1d), which was significantly stiffer compared to the other regions (Fig. 1e, *P*≤0.01, Tukey–Kramer *post hoc* test, *n*≥90 measurement points in each region, Supplementary Table 1). A Tukey–Kramer multi-comparison test of the stiffness maps in these regions (Supplementary Table 1) as well as a two dimensional (2D) correlation analysis between the left and the right brain hemispheres (correlation coefficient *r*=0.90) confirmed a near-perfect symmetry of the brain's mechanical properties with respect to the midline.

### Brain tissue softens after stab injury

**Figure 2:**
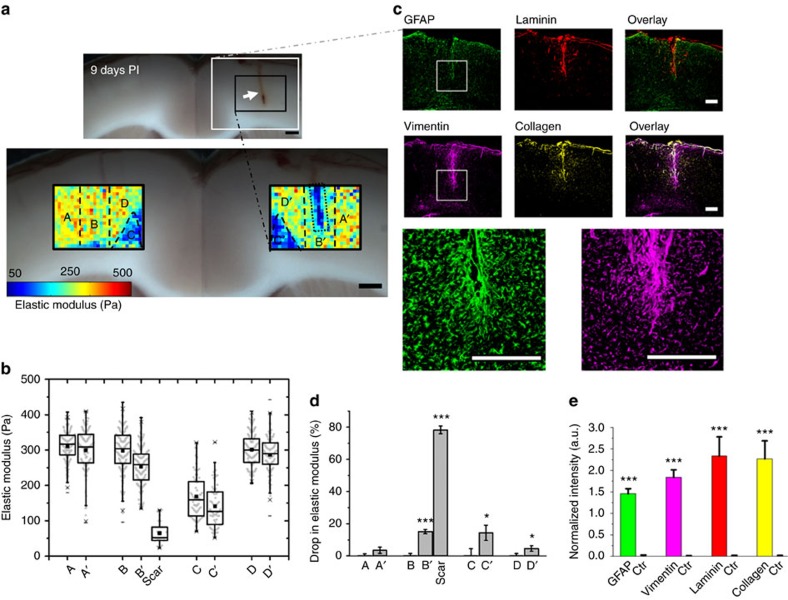
Changes in brain tissue stiffness and protein expression 9 days after a stab injury to the cortex. Cf. Supplementary Fig. 1 for a later time point. (**a**) A 2 mm stab injury (white arrow in the top brightfield image) was induced in the cortex of the rat brain. The colour maps represent the spatial distribution of elastic moduli in the injured and contralateral hemispheres 9 days PI. Five regions were identified for further quantification, including a rectangular region (dashed box) around the injury site (∼150 μm width centred at the scar). (**b**) Comparison of the elastic properties of these regions. (**c**) Representative immunofluorescence images showing that GFAP (green) and vimentin (magenta) are upregulated 9 days PI. Similarly, the ECM proteins laminin (red) and collagen (yellow) are upregulated in and around the site of stab injury. Scale bars, 500 μm. (**d**) Average relative drop in elastic modulus of the regions indicated in (**a**) compared to the uninjured contralateral hemisphere. (**e**) Quantification of immunofluorescence for glial cell and ECM markers. GFAP, vimentin, laminin and collagen IV are all significantly upregulated around the site of injury compared to the contralateral cortical hemisphere. The normalized intensity was derived by comparing the average intensity signal of each marker in a 1.5 × 1.5 mm^2^ square around the scar as indicated in (**c**) with their respective contralateral regions. Error bars are s.e.m., *P<0.01, ***P<0.001.

Having characterized the elastic properties of acute coronal slices of the healthy rat brain, we next measured spatiotemporal changes in tissue elasticity following a controlled sterile unilateral stab injury to the neocortex of the brain. AFM elasticity maps of brain regions near the site of injury and corresponding regions on the contralateral hemisphere, which served as internal control maps, were generated at two different time points post-injury (PI) (Fig. 2a; Supplementary Fig. 1).

AFM stiffness maps of the actual scar regions (defined as a ∼150 μm wide region around the site of injury, Fig. 2a) revealed a drastic drop in tissue elasticity by more than three-fold at 9 days PI (Fig. 2b) compared to the contralateral side. At 22 days PI, we still found an approximately two-fold drop in elastic modulus of the actual scar region (Supplementary Fig. 1b). Moreover, significant tissue softening was measured up to 1,000 μm away from the lesion site (Fig. 2b; Supplementary Fig. 1b). Both at 9 and 22 days PI, the cortical tissue ∼400 μm to either side of the stab injury (regions B′ in Fig. 2a and Supplementary Fig. 1a excluding the actual scar site) was significantly softer than the corresponding areas in the contralateral hemisphere (15% and 13% drop at 9 and 22 days PI, respectively, *P*<0.0001, two-tailed Student *t*-test, *n*≥80 measurement points in each region, Fig. 2d and Supplementary Fig. 1d). Similarly, the medial agranular cortex containing myelinated axon fibres of the cingulum bundle (regions C′ in Fig. 2a and Supplementary Fig. 1a) showed a significant drop in elasticity at both time points (14% drop for 9 days PI, *P*=0.005 and 13% drop for 22 days PI, *P*=0.0008, two-tailed Student *t*-test, *n*≥60 measurement points in each region, Fig. 2d and Supplementary Fig. 1d). Softening of tissue around the cingulum bundle also extended to more dorsal areas of the AGm (regions D′ in Fig. 2a and Supplementary Fig. 1a), with greater decreases measured after 22 days (5% drop for 9 days PI, *P*=0.009 and 15% drop for 22 days PI, *P*=2E-08, two-tailed Student *t*-test, Fig. 2d and Supplementary Fig. 1d). However, at 9 days PI in the lateral agranular cortex (region A′, Fig. 2a and Supplementary Fig. 1a) no significant drop in elasticity was observed (4%, *P*=0.12, two-tailed Student *t*-test, *n*≥100 measurement points in each region, Fig. 2d), whereas a significant decrease was observed after 22 days (11%, *P*=0.002, two-tailed Student *t*-test, *n*≥100 measurement points in each region, Supplementary Fig. 1d).

### Region-specific tissue softening is a robust feature

**Figure 3:**
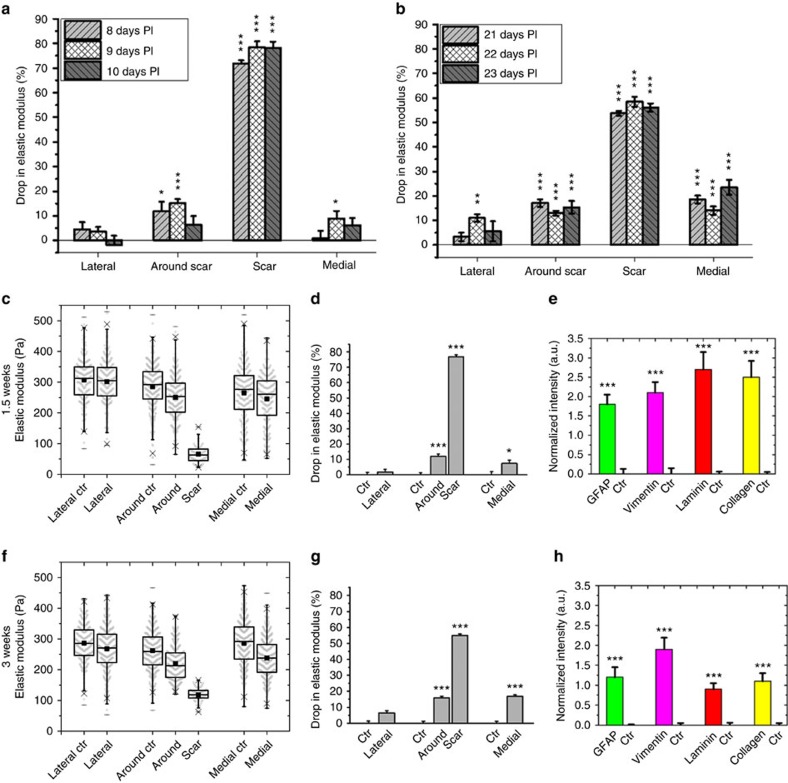
Softening of the brain in response to stab injury. (**a**,**b**) Comparison of the drop in elastic modulus of different brain regions relative to the contralateral control for individual animals at (**a**) ∼1.5 weeks PI and (**b**) ∼three weeks PI. Spatiotemporal changes in brain tissue stiffness were very similar between different animals (Supplementary Tables 2, 3), allowing the data to be pooled. (**c**–**h**) Comparison of the mechanical properties and protein expression of injured and contralateral control tissue at 1.5 weeks PI (**c**–**e**) and three weeks PI (**f**–**h**) (combined data from three animals for each time point). (**c**,**f**) Similar to Fig. 2a, four regions were considered for analysis: region A' (∼500 × 1500 μm^2^ lateral to the scar), region C′+D′ (∼700 × 1500 μm^2^ medial to the scar), region B′ (∼600 × 1500 μm^2^ around the scar excluding the actual scar) and the actual scar region (∼150 × 1500 μm^2^ around the scar). (**d**,**g**) Regional average relative drop in elastic modulus. Tissue was significantly softer at the (Scar), around (B′), and medial (C′+D′) to the scar. (**e**,**h**) The glial cell markers GFAP and vimentin and the ECM markers laminin and collagen were all significantly upregulated around the site of injury both at 1.5 and three weeks PI. Error bars are s.e.m., *P<0.01, ^**^P<0.005; ^***^P<0.001.

To verify the reproducibility of the observed changes in local tissue stiffness following controlled mechanical injury, two more animals per time point were studied, corresponding to ∼1.5 weeks PI (that is, 8 and 10 days, Supplementary Fig. 2) and ∼three weeks PI (that is, 21 and 23 days; Supplementary Fig. 3). No inter-animal variability was observed for the control maps of all six animals (*P*>0.01, Tukey–Kramer multi-comparison test, Supplementary Table 2). Furthermore, the spatiotemporal changes in elasticity (compared to the contralateral control tissue) followed very similar patterns in all animals (Fig. 3a,b). At the site of the stab injury, elastic moduli of tissues injured within the same time interval were statistically comparable (Supplementary Table 3, *P*≥0.19). However, the group of animals investigated at ∼1.5 weeks PI showed a significantly higher drop in elastic stiffness than the group at ∼three weeks PI (*P*<0.001, Tukey–Kramer multi-comparison test, Supplementary Table 3). Considering this statistically significant reversal of tissue softening at the injury site, the regional elasticity maps of individual animals were pooled into two different groups of ∼1.5 weeks and ∼three weeks PI.

Examining the combined data for the three animals sacrificed at either ∼1.5 weeks or ∼three weeks PI revealed a dramatic drop in tissue elasticity of 77% and 55%, respectively, at the site of the stab injury (Fig. 3c,d,f,g; Supplementary Table 4, *P*<0.001). The recovery of tissue stiffness at the injury site at three weeks PI was statistically significant compared to 1.5 weeks PI (*P*<0.001, Tukey–Kramer multi-comparison test, Supplementary Table 4). Approximately 150–400 μm away from the injury (around the scar region), the decrease in elasticity was smaller but still significant (12% and 16% at 1.5 and three weeks PI, respectively; Fig. 3c,d,f,g; Supplementary Table 4, *P*<0.001). The continued softening of the tissue from 12% at 1.5 weeks PI to 16% at three weeks PI was also statistically significant (*P*<0.001, Supplementary Table 4). Although no significant change in stiffness was observed lateral to the scar environment, tissue located medially softened significantly and progressively more with time following injury (7% at 1.5 weeks PI, *P*<0.01 and 17% at three weeks PI, *P*<0.001, Tukey–Kramer multi-comparison test, Supplementary Table 4).

### Tissue softening also occurs after spinal cord crush injury

**Figure 4:**
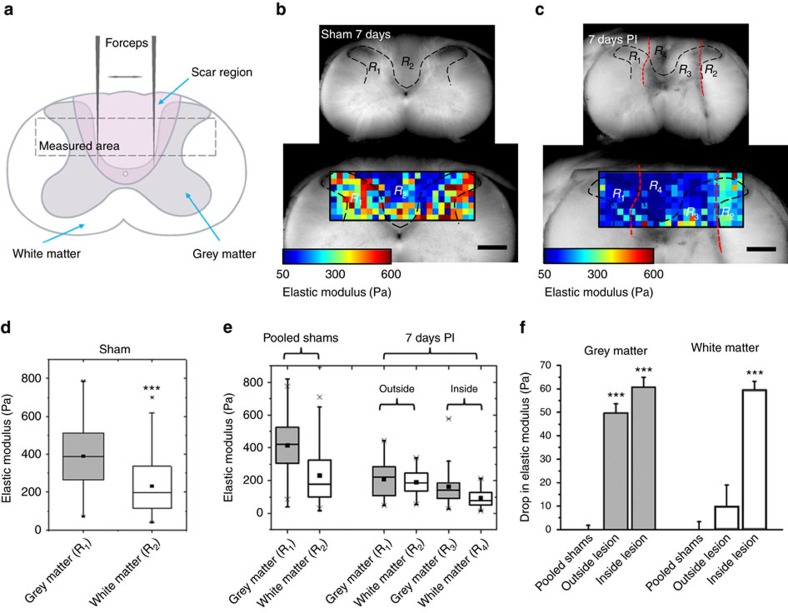
Crush injuries to the rat spinal cord lead to tissue softening. (**a**) Schematic drawing of a dorsal column crush lesion. After a laminectomy at the C5 level, the dura is opened and the spinal cord is penetrated with a pair of sharp forceps (∼1.5 mm deep). Two closures of the forceps for 10 s each crush the dorsal column, creating an injury in both white and grey matter. The dashed rectangle depicts the approximate area in which AFM measurements were performed. (**b**) Transverse spinal cord section of a sham control depicting the outlines of grey (R_1_) and white (R_2_) matter in the area of interest (dashed lines). The colour map represents the spatial distribution of elastic moduli in the grey and white matter. (**c**) Transverse spinal cord section of an animal with a dorsal column crush lesion at 7 days PI. The approximate outlines of the injury are indicated by the red dashed lines. The colour map represents the spatial distribution of elastic moduli in both healthy and injured grey (R_1_ and R_3_, respectively) and healthy and injured white matter (R_2_ and R_4_, respectively). Scale bars, 500 μm. (**d**) Comparison of the elastic properties of grey and white matter in the control animal shown in (**b**). Grey matter was significantly stiffer than white matter (*P*<0.001, two-tailed Student *t*-test). (**e**) Comparison of the elastic moduli of the different tissue regions between four pooled control animals shown in Supplementary Fig. 5 and the tissue shown in (**c**). (**f**) Average relative drop in elastic modulus of the regions indicated in (c) compared to pooled control shams. Error bars are s.e.m., ***P<0.001.

To test whether the observed softening of neural tissue following a stab injury to the brain is specific to either the method of trauma induction or the CNS tissue-type analysed, or if it is a more generalized phenomenon that occurs following different types of injury to the mammalian CNS, we assessed temporal changes in tissue elasticity in a crush injury model of the rat spinal cord (Fig. 4a).

Similar to the cortex, stiffness maps of transversal sections of the healthy spinal cord showed a bilaterally symmetric pattern (Fig. 4b; Supplementary Fig. 6a). The elastic moduli within the white and grey matter were statistically similar in all healthy animals, irrespective of the time point of sham surgery (Supplementary Fig. 5, *P*>0.095, Tukey–Kramer multi-comparison test, Supplementary Table 6). The white matter was always significantly softer than the grey matter (Fig. 4d and Supplementary Fig. 5, *P*<0.001, Tukey–Kramer multi-comparison test, Supplementary Table 6), with grey matter being about twice as stiff as white matter (Fig. 4d,e, median of 420 Pa for pooled grey and 177 Pa for white matter, *P*<0.001, two-tailed Student *t*-test). On the basis of conserved elasticity patterns, all control animals were pooled and grey and white matter investigated separately.

In contrast to the well-defined and very reproducible boundaries of cortical stab lesions, spinal cord crush injuries resulted in irregular and diffuse boundaries with a varying degree of tissue damage among different animals (Fig. 4c; Supplementary Fig. 6b). For the analysis of injured spinal cord elasticity, we defined four tissue regions based on the visual appearance of the crush injury: regions R_1_ and R_2_, corresponding to grey and white matter outside of the visible injury, and regions R_3_ and R_4_, corresponding to grey and white matter within the actual injury (Fig. 4c,e,f; Supplementary Fig. 6). Similar to what we observed for cortical tissue after stab injuries, within the lesion site the stiffness of the spinal cord grey matter was significantly lower than in control tissue in 5 of 6 animals (*P*<0.001 except one animal at 8 days PI with *P*=0.083, Tukey–Kramer multi-comparison test; Fig. 4e,f , Supplementary Fig. 6, Supplementary Table 7). In 2 of 3 animals at ∼1.5 weeks PI and in 1 of 3 animals at ∼three weeks PI, grey matter also significantly softened outside the visible lesion (Fig. 4e,f, Supplementary Fig. 6 and Supplementary Table 7). In addition, white matter significantly softened within the injury site in 2 of 3 animals at ∼1.5 weeks PI (*P*<0.001, Tukey–Kramer multi-comparison test; Fig. 4e,f, Supplementary Fig. 6 and Supplementary Table 8). We observed a greater variability between individual animals compared to cortical stab lesions, likely due to the variable amounts of tissue damage associated with this injury model and a higher level of uncertainty in determining the correct size and location of the lesion. Yet the spinal cord crush injury experiments confirmed that rat CNS tissue softens after different types of traumatic injuries at the post-injury time points studied here.

### Reactive gliosis around the injury site

The precise application of cortical stab injuries led to well-defined tissue boundaries at the injury site and thus enabled us to investigate correlations between the expression of established markers of glial scarring and the observed mechanical changes in the rat cortex. Thin cryosections from slices used for AFM measurements were immunolabelled using antibodies targeting the glial intermediate filament proteins, GFAP and vimentin, as well as the extracellular matrix molecules, laminin and collagen IV (Fig. 2c and Supplementary Fig. 1c).

All four proteins were significantly upregulated by more than two-fold around the scar at 9 and 22 days PI as compared to the corresponding region on the contralateral hemisphere (Fig. 2e and Supplementary Fig. 1e, *P*<0.001, 12 ROIs in two slices, two-tailed Student *t*-test). GFAP and vimentin levels dropped slightly but not significantly at 22 days compared to 9 days PI (*P*>0.01, two-tailed Student *t*-test, Supplementary Table 5) which may explain the prolonged levels of tissue softening around the scar (region B′ in Fig. 2a and Supplementary Fig. 1a). In contrast, levels of both extracellular matrix proteins, while still significantly higher than in control tissue, were significantly lower on day 22 PI compared to 9 days PI (*P*<0.005, two-tailed Student *t*-test, 12 ROIs in two slices, Supplementary Table 5), indicating that the glial scar tissue had partially healed within the first three weeks PI in close proximity to the injury site. This observed cortical wound healing also coincided with an increase in the actual scar tissue elasticity from 1.5 to three weeks towards baseline levels (Fig. 2b, Supplementary Fig. 1b, and Fig. 3c,f). Combined fluorescence intensity analyses for brain slices imaged at 1.5 and three weeks PI followed very similar trends as those described for 9 and 22 days PI (Fig. 3e,h).

**Figure 5:**
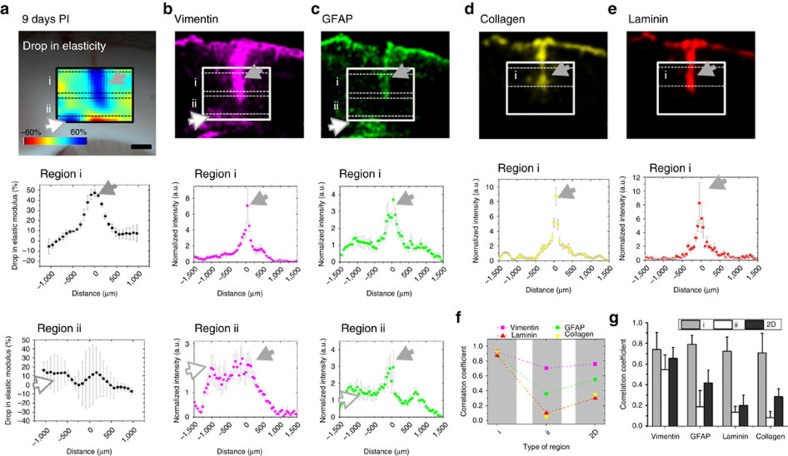
Correlation between brain tissue mechanics, gliosis, and ECM changes at 9 days PI. Cf. Supplementary Fig. 4 for a later time point. (**a**) Rainbow-pallet map showing the relative difference in elastic modulus between injured and uninjured cortical hemisphere. Scale bar, 500 μm. The panels in the bottom rows represent vertical projection profiles (mean±s.d.) of the changes in elasticity determined for the regions i and ii indicated by dashed lines in the map . (**b**,**c**) The normalized and down-scaled pixel intensity image of vimentin and GFAP expression. The panels in the bottom rows represent vertical projection profiles (mean±s.d.) of the vimentin and GFAP normalized intensity estimated for the regions i and ii indicated by dashed lines in their respective images. (**d**,**e**) The normalized and down-scaled pixel intensity image of collagen IV and laminin expression. The panels in the bottom row represent vertical projection profiles (mean±s.d.) of the collagen and laminin normalized intensity in region i of their respective fluorescence images. In (**a**–**e**), grey arrows indicate the direct positive correlation between a drop in elasticity and an increase in protein expression. A positive correlation between tissue softening and vimentin and GFAP expression was also observed in regions located far away from the injury site medial to the scar (white arrows). (**f**) Shown are the calculated linear correlation coefficients for regions i and ii as well as the 2D correlation coefficients derived by linearly correlating the 2D matrix of the change in elasticity and the 2D maps of fluorescence intensity for each marker. (**g**) Average (mean±s.d.) correlation coefficients of three brains at 9, 21 and 22 days PI as a function of protein type and correlation region.

**Figure 6:**
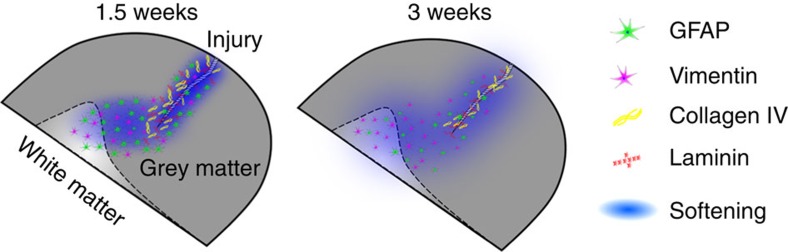
Schematic summary. 1.5 weeks after injury to the rat cortex, brain tissue has significantly softened. Softening is mostly restricted to the site of injury, where collagen IV, laminin, GFAP and vimentin are upregulated. At later time points (that is, at three weeks PI), scar tissue elasticity somewhat recovers at the stab injury site but mild tissue softening spreads away from the scar, which strongly correlates with the dispersion of an increased number of vimentin-expressing cells.

Projection profiles of the fluorescence markers revealed that, while laminin and collagen IV upregulation was limited to a ∼250 μm wide region (calculated from the full width at half maximum, FWHM) around the stab injury site 9 days PI (Fig. 5d,e), GFAP and vimentin expression levels were increased in regions >1,000 μm away from the stab wound, with increased expression levels propagated mainly towards the midline (Fig. 5b,c). Glial cell reactivity was particularly evident in the cingulum region, which contains a relatively high proportion of myelinated neurons (area C′ in Fig. 2a and white arrows in Fig. 5b,c). As described above, the cingulum also displayed a greater drop in elasticity compared to the predominantly grey matter region of the medial agranular cortex (area D′ in Fig. 2a; 14 versus 4% in Fig. 2d), which showed less reactive gliosis. At 22 days, the width of laminin and collagen IV upregulation had narrowed dramatically to a region of ∼100 μm radius (calculated from the FWHM) around the stab injury (Supplementary Fig. 4d, e), and asymmetric upregulation of GFAP towards the midline was reduced but still present (Supplementary Fig. 4c), while the scattering of vimentin upregulation was maintained (Fig. 6; Supplementary Fig. 4b).

### Positive correlation between gliosis and tissue softening

Because patterns of glial cell activation and tissue softening seemed to overlap, we investigated whether there was a direct spatial correlation between tissue softening and the expression of glial intermediate filament or ECM protein markers. To perform a precise correlation analysis, we only considered samples for which AFM and immunofluorescence measurements could be done on slices of the very same plane, which restricted our analysis to three samples from 9, 21 and 22 days PI. We combined the elasticity map around the scar with its corresponding map in the contralateral hemisphere to obtain a ratiometric field that represents the relative drop in elastic modulus (that is, a proportionate tissue softening map, Fig. 5a and Supplementary Fig. 4a). We similarly normalized the immunofluorescence images and scaled them down to obtain expression maps of gliosis markers with the same pixel size (100 × 100 μm^2^) as the tissue softening maps (Fig. 5b–e; Supplementary Fig. 4b–e).

To analyse the spatial correlation between the local fluorescence signal and tissue stiffness, we calculated the average vertical projection of the softening and normalized the intensity maps in two stripes covering the scar (i and ii in Fig. 5 and Supplementary Fig. 4). The vertical projection profiles of tissue softening in the first stripe (i) showed a strong positive correlation with the vertical projection profiles of all markers in all three investigated animals (*r*=0.74±0.16 for vimentin, 0.79±0.08 for GFAP, 0.72±0.13 for collagen and 0.71±0.19 for laminin, mean±s.d., Fig. 5g and Supplementary Fig. 4), with the highest correlations for all markers at 9 days PI (*r*>0.8, Fig. 5f) compared to 21 and 22 days PI (Supplementary Fig. 4f,g). However, in the lower stripe (ii), which overlaps with myelinated fibres of the cingulum and AGl regions away from the brain midline, only vimentin showed a strong positive correlation with tissue softening (Fig. 5f and Supplementary Fig. 4f, average of *r*=0.55±0.15, Fig. 5g). In this region, GFAP levels displayed some moderate correlation with tissue softening at 9 days PI (*r*=0.36, Fig. 5f), while at later stages GFAP as well as ECM markers were only weakly correlated with tissue softening (*r*<0.3, Supplementary Fig. 4f,g). Finally, a two dimensional correlation analysis between protein expression maps and tissue softening revealed that vimentin had the strongest correlation with softening of the tissue at all stages following the injury (*r*=0.65±0.1, Fig. 5g).

## Discussion

In this study, we measured the mechanical properties of the adult rat brain cortex and spinal cord in normal conditions and in response to two different types of traumatic injury using AFM. Healthy CNS tissue is mechanically heterogeneous; we found grey matter to be about twice as stiff as white matter in both brain and spinal cord, which is consistent with recent findings showing that white matter is softer than grey matter also in the human brain, rat cerebellum, and in the mouse spinal cord^31^,^32^,^33^. While our measurements showed that the rat cortex is very soft, it is still slightly stiffer than embryonic mouse cortex^34^. Similarly, spinal cord tissue of rats is about twice as stiff as that of mice^32^. Whereas these differences in mechanical properties could be mainly due to species-specific differences, it is possible that the age of the animals also influences overall brain tissue stiffness^34^,^35^.

Scar tissue in the body is usually stiffer than the surrounding healthy tissue^2^,^3^,^4^,^5^,^6^,^7^,^8^,^9^,^10^. On the basis of the assumption that glial scars are similarly stiffer than healthy CNS tissue, it is a long-standing and widespread assumption that glial scars not only pose a biochemical but also a mechanical barrier to neuronal regeneration^15^,^16^,^17^. Here we show that, similar to tissue that replaces CNS tissue after spinal cord hemisections^36^, neural tissue itself in both the rat brain cortex and spinal cord does not stiffen but rather becomes softer after injury. While the softening of tissue was pronounced mainly in the immediate vicinity of the injury, it also spread towards more distant regions (Fig. 3 and Supplementary Fig. 6; summarized for cortical tissue in Fig. 6). The observed decrease in cortical tissue stiffness after stab injury correlated in all three investigated tissue sections with an increased expression of vimentin, GFAP, laminin and collagen IV.

The largest drop in cortical elasticity was observed in the immediate vicinity of the injury (that is, injury site and B′ regions in Figs 2 and 3), where both types of glial intermediate filaments and ECM components were upregulated. Although at later stages the elastic modulus of cortical tissue remained significantly softer than that of the contralateral control tissue, it partially recovered toward pre-injury levels between days 9 and 23 PI in that narrow region (Fig. 3a,b and Supplementary Table 4). Concomitantly, expression levels of laminin and collagen IV also reversed near the injury site and decreased significantly (Fig. 3e, h and Supplementary Table 5), suggesting that these ECM components, which are part of basement membrane, contribute to glial scar elasticity.

However, the lower but still clearly significant decrease in cortical tissue stiffness at greater distances from the injury site (that is, 500–1,000 μm, corresponding to regions A′, C′ and D′ in Fig. 2), particularly in those regions containing myelinated fibres of the cingulum (regions medial to the scar, Fig. 3 and Supplementary Table 4), was mostly accompanied by an upregulation of glial intermediate filaments (Fig. 5b, c and Supplementary Fig. 4b, c). While in our experiments increased GFAP expression correlated well with a decrease in cortical tissue elasticity (particularly at 9 days PI, Fig. 5f), the distribution of vimentin upregulation was an even better predictor of decreased tissue elasticity in the penumbra region of the glial scar, both at early and late stages (Fig. 5f,g and Supplementary Fig. 4f,g). The observed increase in GFAP levels suggests that long range signalling activated distant glial cells, which might be an attempt to facilitate regenerative processes^37^. An increase in vimentin levels, on the other hand, which in the brain is considered a marker of immature glia and new-born neural stem cells, could be related to the proliferation of those cell types^38^,^39^.

The softening of cortical glial scars can be partly explained by the specific composition of the ECM, whose expression is upregulated after injury^40^,^41^. The ECM in scars outside the CNS is usually rich in collagen-1, which scales with tissue stiffness^1^. In the investigated types of glial scars, collagen-1 is largely absent^30^, which could partly explain the softness of the tissue. Furthermore, glial scar ECM is dominated by proteoglycans, such as CSPGs, which are upregulated around the injury site^18^, highly hydrated, and could thus contribute to further local tissue softening^27^. Collagen IV and laminin are mainly associated with basal laminae^42^. Their initial upregulation, which was associated with a decrease in tissue elasticity, was likely a sign of the destruction of blood vessels. The reformation of the blood–brain barrier could thus contribute to readjusting CNS tissue elasticity towards its normal state at later stages.

Another factor that is likely contributing to the opposite mechanical behaviour of scars in the brain and the spinal cord compared to other body tissues is the cellular composition. Myofibroblasts, which are found in large numbers in fibrotic body tissue and scars, are not just significantly stiffer than glial cells^43^,^44^ but also highly contractile. The strain they apply on their environment leads to a stiffening of the tissue surrounding them^45^, thus likely contributing to the overall enhanced stiffness of the scars. Glial scars, in contrast, mainly consist of activated glial cells, pericytes, and meningeal cells^11^. *In vitro*, while vimentin is a contributor to cell mechanics^46^, an increase in GFAP levels in cortical astrocytes following a mechanical stretch injury is accompanied by a softening of the cells as measured by AFM (ref. 47). Thus, glial cells in the injured rat cortex could be similarly activated and softened.

Finally, spinal cord injuries are accompanied by a loss of cholesterol and lipids as determined by Raman spectroscopy^48^, partly due to demyelination of damaged axons. The degree of myelination, on the other hand, scales with CNS tissue stiffness^49^,^50^. Hence, the loss of myelin might be another contributor particularly to the softening of the white matter after injuries to both rat cortex and spinal cord (Figs 2, 3, 4 and Supplementary Fig. 6).

During CNS development and regeneration, axons move through and mechanically interact with a highly complex environment^19^,^20^,^21^,^27^,^51^,^52^. During this growth phase, they detect and respond to a variety of signals, including tissue stiffness. Axons of dorsal root ganglion cells, for example, grow better on stiffer substrates *in vitro*^20^, and *Xenopus* retinal ganglion cell (RGC) axons similarly grow faster and are more directionally persistent in stiffer environments^27^. When exposed to stiffness gradients, RGC axons preferentially grow towards the soft side of the tissue *in vitro* as well as *in vivo*^27^, in contrast to microglial cells, which migrate towards the stiff side^24^. Both changes in brain tissue stiffness and perturbed mechanosensing lead to aberrant RGC axon growth patterns with severe pathfinding errors^27^, suggesting an important contribution of mechanical signals to regulating axon growth. After CNS injuries in adult mammals, neurons usually do not regrow, which could thus at least partly be a consequence of altered mechanical signals in their environment, that is, the softening of the tissue.

CNS tissue responds to injury, amongst others, by the proliferation of resident stem cells. The lineage choice in human neural stem cells can be directed by mechanical signals: on soft substrates, they give rise to glial cells, while on stiff substrates they preferentially differentiate into neurons^53^. Hence, the softening of mammalian CNS tissue after injury might not only directly provide an inhibitory signal for neuronal regeneration by limiting axon growth but also by tuning the stem cell fate towards glial cell lineages.

As an indirect effect of the altered mechanical signals, the enhanced number of activated glial cells and pericytes may then secrete more molecules inhibitory to axon regeneration, such as CSPGs. Also, the breakdown of myelin, which is generally associated with injuries to CNS tissue, may not only contribute to the softening particularly of the myelinated fibres of the cingulum^49^,^50^ but also have a negative effect on neuronal regeneration through the exposure of molecules such as Nogo^54^.

It is intriguing to speculate whether glial scars in vertebrates that are capable of regenerating damaged CNS neurons, such as Urodeles (that is, newts, salamanders and axolotls), are also softer or rather stiffer than healthy tissue, as an enhanced stiffness might facilitate neuronal regeneration. Nevertheless, scars in the rat cerebral cortex and spinal cord have a soft mechanical signature following stab and crush injury; a property that appears unique to the CNS. Providing appropriate mechanical signals in addition to permissive chemical signals^28^,^37^ should be considered in future approaches in regenerative medicine and in the design of neural implants to ultimately facilitate functional recovery after CNS injuries.

## Methods

### Animals and surgical procedures

All experimental procedures involving animals were carried out in accordance with the UK Animals (Scientific Procedures) Act 1986. All surgeries were performed using aseptic techniques. Eight weeks old female Lister hooded rats (Harlan UK) were socially-housed in groups of 4 and given *ad libitum* access to food and water. At post-natal day 90, a cortical stab injury to the left cerebral cortical hemisphere was performed to rats under anaesthesia. Briefly, animals were anesthetized with isoflurane (4% in O_2_ for induction) and maintained at 1.5–2% in O_2_ delivered via a facemask. Body temperature was monitored via a rectal thermometer and maintained at ∼36 °C with a heating pad. Animals were transferred to a stereotaxic frame (David Kopf Instruments) where the scalp was shaved and the skin was disinfected with alcohol. A midline incision was made and a craniotomy was performed to create one linear hole over the left cerebral hemisphere at the following coordinates, defined as anterioposterior (AP), mediolateral (ML): +2 mm to −1 mm AP; 2 mm ML, relative to Bregma. The dura mater was incised and a sterile micro-blade (15° angled blade; 1 mm blade thickness) was inserted at a depth of 2 mm into the sensorimotor cortex and cut from the same coordinates listed above (from +2 mm to −1 mm AP; 2 mm ML relative to Bregma) (Fig. 1a). The site of injury was then covered with sterile bone wax, the scalp was sutured and the rat was allowed to recover in its cage for a minimum of 9 days. For spinal cord dorsal column crush injuries, 12 weeks old female Lister hooded rats (Charles River) were socially-housed in groups of 3 and given *ad libitum* access to food and water. Animals were anesthetized with 5% isoflurane and maintained at 1.5–2% isofluorane in 1.5–2.0 l min^−1^ oxygen and analgesics were given. Body temperature was monitored via a rectal thermometer and maintained at ∼37 °C with a heating pad. A laminectomy was performed at the C5 level. The dura was pierced at two locations and the dorsal columns were crushed with fine forceps at a depth of 1.5 mm (Fig. 4a). Because the spread of crush lesions in the spinal cord is less controlled than in stab injuries in the cortex (allowing for internal controls on the contralateral brain side), separate control animals had to be used, which underwent laminectomy but no dura opening or crush. The site of injury was covered with gelfoam before the muscle layers and skin were sutured. Animals recovered in an incubator (37 °C) and were subsequently transferred back into their home cages. All animals were given analgesics for 4 days following surgery.

### Dissection and preparation of tissue

At ∼1.5 weeks (7, 8, 9 and 10 days) or ∼three weeks (20, 21, 22 and 23 days) post-surgery, rats were anesthetized with 5% isoflurane and sacrificed by intraperitoneal (i.p.) injection of a lethal dose of pentobarbitone sodium (Euthatal) and subsequent decapitation (brain) or permanent cessation of the circulation confirmed by subsequent cardiac perfusion with cold (4 °C) slicing artificial cerebrospinal fluid (aCSF; spinal cord). The tissue was dissected out and kept in cold slicing aCSF for 5 min. The composition of slicing aCSF was: 191 mM sucrose, 0.75 mM K-gluconate, 1.25 mM KH_2_PO_4_, 26 mM NaHCO_3_, 4 mM MgSO_4_, 1 mM CaCl_2_, 20 mM glucose, 2 mM kynurenic acid, 1 mM (+)-sodium L-ascorbate, 5 mM ethyl pyruvate, 3 mM myo-inositol, and 2 mM NaOH (ref. 32). For cortical sections, the cerebellum and olfactory bulbs were then removed with a sharp blade and the brain was glued onto a vibratome platform (Leica VT1000 S). Coronal slices of the brain were cut (Fig. 1a) in cold slicing aCSF until the glial scar was reached. Then, a 2 mm thick coronal slice of the brain was cut and placed into a 35 mm glass-bottom petri dish and submerged in cold slicing aCSF solution. For spinal cord sections, a ∼1 cm long piece of spinal cord covering the injury site was freed from the overlying dura and embedded in 4% low melting point agarose (Sigma Aldrich; in PBS)^32^. A small agarose block containing the tissue was then glued onto a vibratome platform and 500 μm thick transverse sections were cut in cold slicing aCSF bubbled with 95% O_2_ and 5% CO_2_. Sections were collected and placed in a 35 mm dish treated with BD Cell-Tak (Cell and Tissue Adhesive; BD Biosciences). The samples were transferred to an inverted microscope and perfused with fresh measuring aCSF (121 mM NaCl, 3 mM KCl, 1.25 mM NaH_2_PO_4_, 25 mM NaHCO_3_, 1.1 mM MgCl_2_, 2.2 mM CaCl_2_, 15 mM glucose, 1 mM (+)-sodium L-ascorbate, 5 mM ethyl pyruvate and 3 mM myo-inositol) bubbled with 95% O_2_ and 5% CO_2_ at a flow rate of 20 ml per h. The dishes were slowly brought to room temperature for 15–30 min before AFM measurements commenced.

### Atomic force microscopy

AFM indentation measurements were performed with a JPK Nanowizard Cellhesion 200 (JPK Instruments AG, Berlin, Germany) placed on an inverted optical microscope (Axio Observer.A1, Carl Zeiss Ltd., Cambridge, UK) and a motorized xy stage. For AFM measurements of cortical sections and spinal cord slices, cantilevers with pyramidal tips (MLCT, Bruker, nominal spring constant of 0.1 N m^−1^) and tipless silicon cantilevers (Arrow-TL1, NanoWorld, Neuchatel, Switzerland, spring constants ∼0.07 N m^−1^) were used, respectively. Cantilevers were modified by gluing 89.3 μm diameter polystyrene beads (microParticles GmbH, Germany) to the tip of the cantilever via ultraviolet curing glue (ultraviolet curing, Loctite). Cantilever spring constants were determined using the thermal noise method implemented in the AFM software (JPK SPM). A CCD camera (The Imaging Source, Bremen, Germany) was mounted on top of the AFM setup to align and monitor the position of the cantilever over defined regions of the brain slice. Force–distance curves were taken with an approach speed of 5–10 μm s^−1^ and a set force of 20 or 30 nN by an automated raster scan using the motorized stage with 75 or 100 μm step size for brain or spinal cord tissue slices. Using the method described in^55^,^56^ the contact point was found and subsequently the indentation depth *δ* was calculated by subtracting the cantilever deflection d from the piezo translation z after contact *δ*=*z*−*d*. The elastic moduli were extracted from the force–distance curves by fitting the contact portion of curves to a Hertz contact model between a sphere and an infinite half space^57^ using a MATLAB (Mathworks) routine previously described^31^,^58^. The boundaries of the lesion were determined by eye. For the segmentation of regions of interest and pooling of data we used custom-written MATLAB routines^27^.

Taking the size of the spherical indenter into account, the applied maximum force was chosen to probe the tissue stiffness on a cellular length-scale while also adhering to small strain conditions^55^,^59^. Setting the maximum force to 20–30 nN produced average indentation depths δ∼4 μm and maximum δ∼15 μm (for the softest parts of the tissue). Limiting the analysis of force–indentation curves to a maximum indentation depth of 10 μm resulted in a contact radius 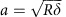 ∼20 μm. Under these assumptions, the small indentation strain condition of 0.2 × *a*/*R*<0.02 and *a*/*h*<<0.1, where *R* is the radius of the indenter and *h* the slice thickness, was valid^60^ and the Hertz formulation yields a good approximation to extract mechanical properties of CNS tissue^61^. However, a small but noticeable deviation from the exponent of the Hertz model was apparent in the log–log plots of normalized force–indentation curves (Fig. 1b). Different sources of nonlinearity such as the experimental condition of large deformations and the inherent nonlinearities associated with soft tissues may contribute to this deviation^62^. We minimized geometrical nonlinearities by using fairly large indenters and limiting the indentation depth.

### Immunofluorescence and fluorescence microscopy

The 2 mm coronal brain slices used for AFM were fixed in 10% formalin solution for 6–8 h at 4 °C and then transferred to 30% sucrose solution overnight (∼18 h at 4 °C) for cryoprotection. Fixed brain slices were then covered in optimal cutting temperature compound (OCT), frozen and stored at −80 °C until cryosectioning was performed. Using a cryostat (Leica), 14 μm sections were adhered to glass slides coated with poly-L-lysine solution (0.1% (w/v) in H_2_O, Sigma). Sections were permeabilized in a solution of 0.1% Triton X-100 (Sigma) in phosphate buffered saline (PBS) for 25 min. They were then blocked in 5% bovine serum albumin (BSA, Sigma) for 1 h followed by incubation overnight (18 h) in primary antibody solution containing 1% BSA in PBS. The sections were immunofluorescently-labelled for several cell and extracellular matrix markers including: (1) glial fibrillary acidic protein (GFAP) using a chicken polyclonal antibody (Abcam ab4674, 1:2,000 dilution); (2) vimentin using a chicken polyclonal antibody (Abcam ab24525, 1:200 dilution); (3) laminin using a rabbit polyclonal antibody (Abcam ab11575, 1:200 dilution); and (4) collagen IV using a rabbit polyclonal antibody (Abcam ab6586, 1:200 dilution). The primary antibodies were then washed in PBS (3 × 15 min) and sections were incubated for 4 h with donkey anti-chicken IgY (H+L) CF 633 (Sigma SAB4600127, 1:1,000 dilution) and donkey anti-rabbit IgG (H+L) Alexa Fluor 488 (Abcam ab150061, 1:1,000 dilution) secondary antibodies. The sections were then washed with PBS (3 × 15 min) and mounted with a glass coverslip (no.1 thickness, Menzel-Gläser, Germany) using ProLong Gold Antifade Mountant with DAPI (Life Technologies, UK). Microscope slides were left to cure in darkness at room temperature for 24 h and then stored at 4 °C until image capture was performed.

Fluorescence images used for quantitative analysis were captured with an Axio Scan.Z1 slide scanner (Zeiss, Germany). Z-stack images (five z-slices per field of view) were captured using a 20 × magnification objective (Plan-Apochromat) and images of the maximum intensity projections were stitched using ZEN software. For quantitative fluorescence intensity analysis, uniform microscope settings were maintained throughout all image capture sessions.

### Quantification and statistical analysis

Statistical analyses and plotting were performed in MATLAB or Origin (OriginLab). For the maps showing relative differences in tissue stiffness, the ipsilateral and contralateral stiffness maps (as shown in Fig. 2a) were first smoothed via a 2D average filter (size of 3 × 3 pixels or ∼250 × 250 μm^2^) and then the smoothed maps were subtracted from each other. Normality of distributions was tested using the Lilliefors test and the difference between two groups was evaluated by a two-tailed Student's *t*-test. A Mann–Whitney–Wilcoxon test was employed where normality could not be assumed. The differences between multiple groups were tested by a general linear ANOVA model followed by Tukey–Kramer *post hoc* test to obtain the multiple comparison *P* values. The exact *P* values are reported in the Supplementary Tables; *P* values of < 0.01 were considered statistically significant. In the figures, *P*<0.01 is represented by *, *P*<0.005 by ^**^, and *P*<0.001 by ^***^. In all box plots, the top and bottom of the box represent the 75th and 25th percentiles respectively and the line inside corresponds to the median. The filled square denotes mean and whiskers the range of outer-most data points that fall within 1.5 × interquartile range. The overlaid data points represent the experimental data binned into about 20 equally spaced intervals.

### Data availability

The data that support the findings of this study are available from the corresponding authors on request.

## Additional information

**How to cite this article:** Moeendarbary, E. *et al*. The soft mechanical signature of glial scars in the central nervous system. *Nat. Commun.* **8,** 14787 doi: 10.1038/ncomms14787 (2017).

**Publisher's note:** Springer Nature remains neutral with regard to jurisdictional claims in published maps and institutional affiliations.

## Supplementary Material

Supplementary Information Supplementary Figures and Supplementary Tables
