# Supplementary material for: The soft mechanical signature of glial scars in the central nervous system
Source: Nat Commun. 2017 Mar 20;8:14787. doi: 10.1038/ncomms14787 (PMC5364386; doi:10.1038/ncomms14787)
Supplement: Supplementary Information — Supplementary Figures and Supplementary Tables [file ncomms14787-s1.pdf]

Supplementary material

Supplementary figures

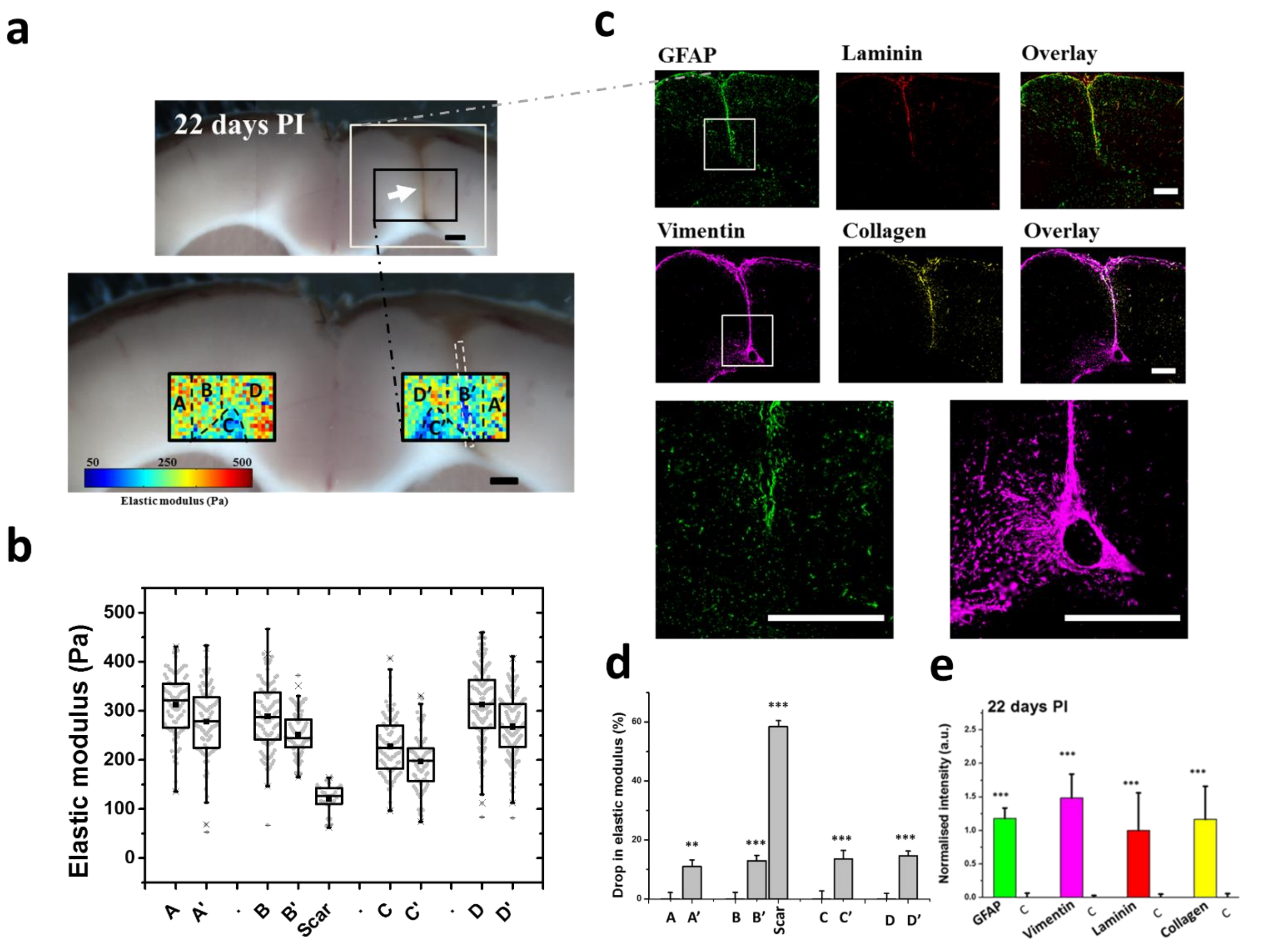

Supplementary Figure 1: **Changes in brain tissue stiffness and protein expression 22 days after a stab injury to the cortex (*cf.* Fig. 2 for an earlier time point). (a)** A 2mm stab injury (white arrow in the top brightfield image) was induced in the cortex of the rat brain. The colour maps represent the spatial distribution of elastic moduli in the injured and contralateral hemispheres 22 days PI. Five regions were identified for further quantification, including a rectangular region (dashed box) around the injury site (~150 μm width centred at the scar). **(b)** Comparison of the elastic properties of these regions. **(c)** Representative immunofluorescence images showing that GFAP (green) and vimentin (magenta) are upregulated 22 days PI. Similarly, the ECM proteins laminin (red) and collagen (yellow) are upregulated in and around the site of stab injury. Scale bars=500 μm. **(d)** Average relative drop in elastic modulus of the regions indicated in **(a)** compared to the uninjured contralateral hemisphere. **(e)** Quantification of immunofluorescence for glial cell and ECM markers. GFAP, vimentin, laminin and collagen IV are all significantly upregulated around the site of injury compared to the contralateral cortical hemisphere. The normalised intensity was derived by comparing the average intensity signal of each marker in a 1.5 X 1.5 mm^2 square around the scar as indicated in **(c)** with their respective contralateral regions. Error bars are SEM, * P<0.01, ** P<0.005, *** P<0.001.

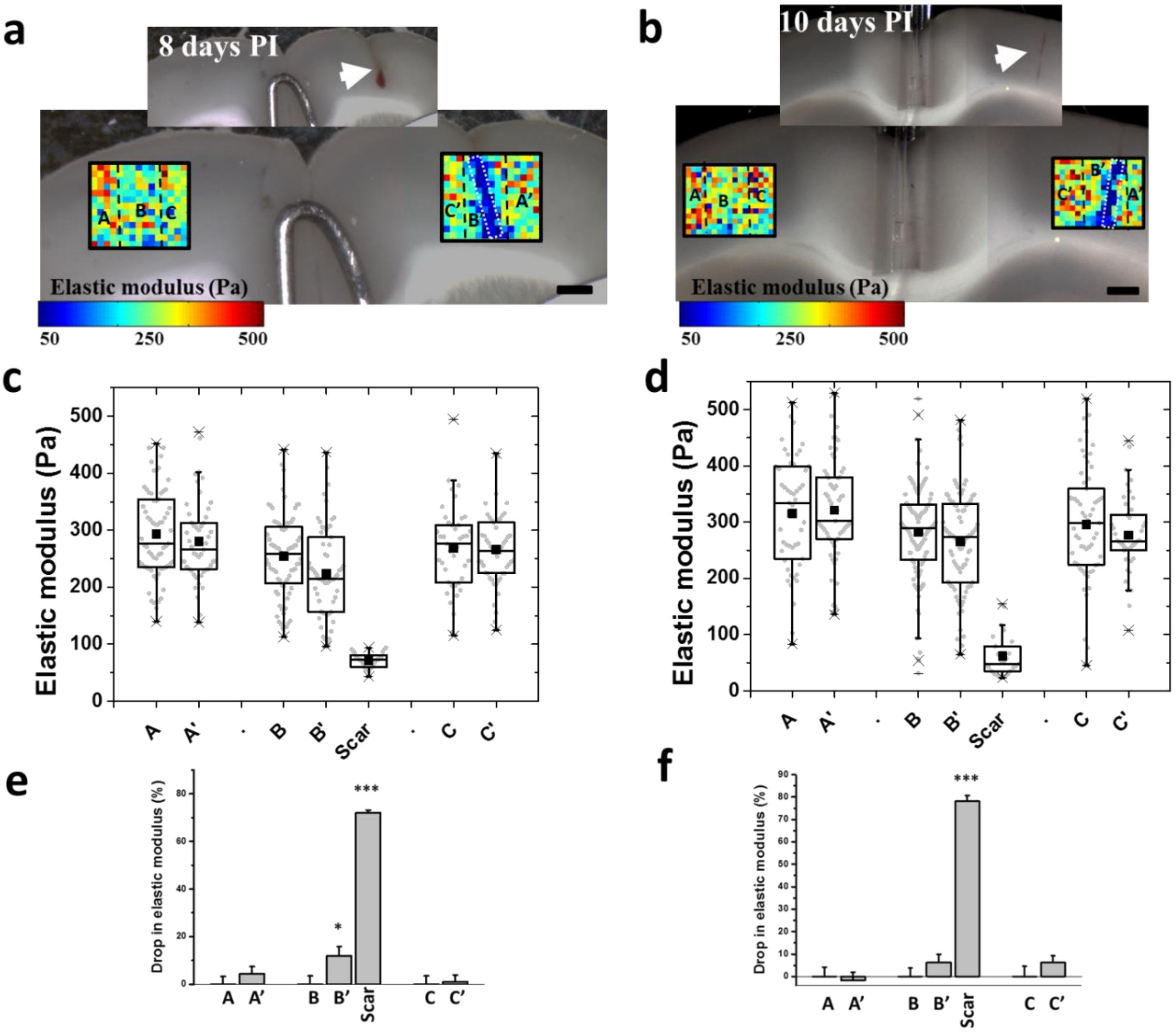

Supplementary Figure 2: Changes in brain tissue stiffness at two further early time points after cortical stab injury (*cf.* Fig. 2). (a, b) A 2mm stab injury (white arrow in the top brightfield image) was induced in the cortex of the rat brain. The colour maps represent the spatial distribution of elastic moduli in the injured and contralateral hemispheres 8 **(a)** and 10 **(b)** days PI. Four regions were identified for further quantification, including a rectangular region (dashed rectangle) around the injury site (~150 μm width centred at the scar). **(c, d)** Comparison of the elastic properties of these regions. **(e, f)** Average relative drop in elastic modulus of the regions indicated in **(a,b)** compared to the uninjured contralateral hemisphere. Error bars are SEM, * P<0.01, *** P<0.001.

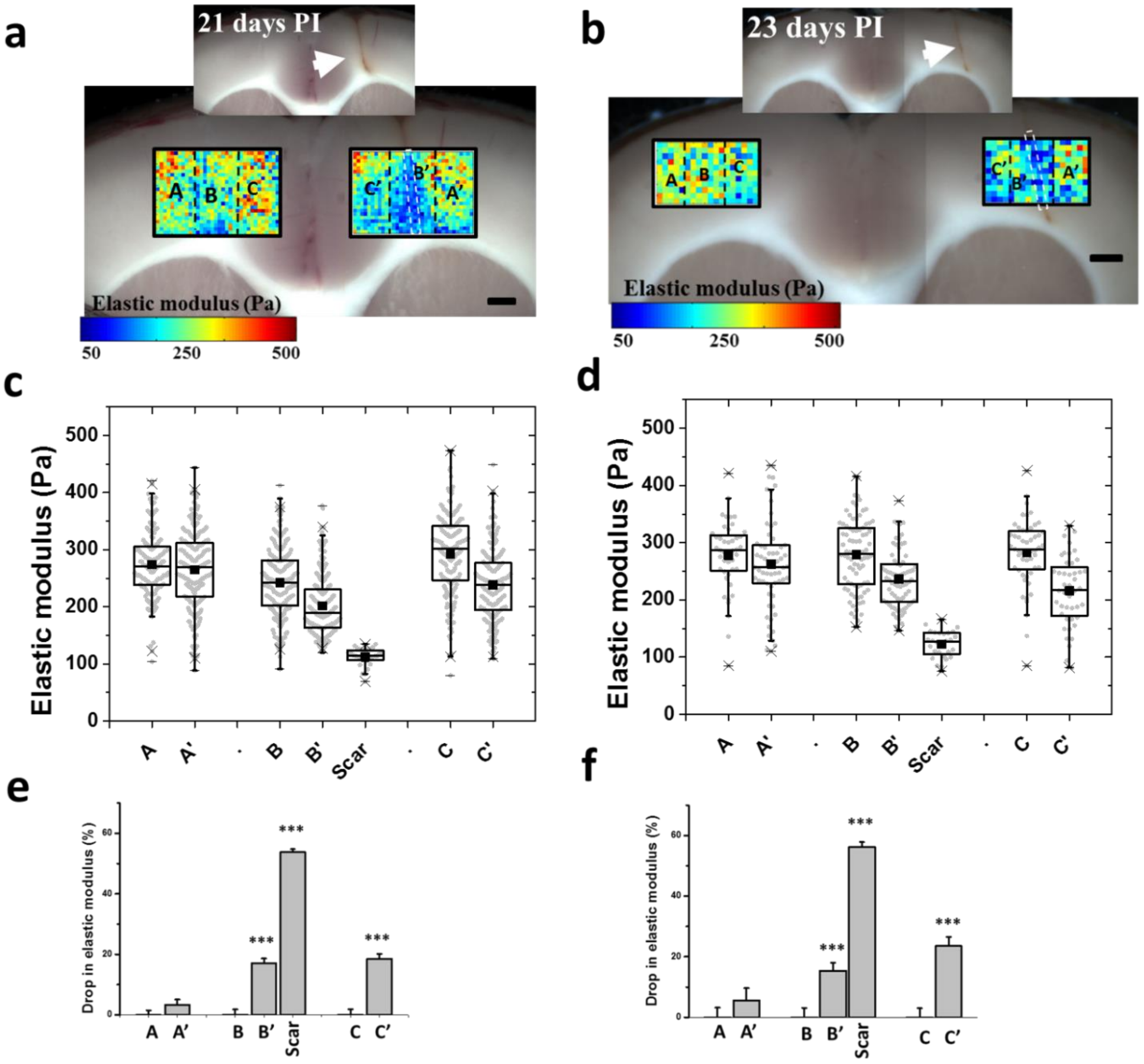

Supplementary Figure 3: Changes in brain tissue stiffness at two further late time points after cortical stab injury (*cf.* Fig. S1). (a, b) A 2mm stab injury (white arrow in the top brightfield image) was induced in the cortex of the rat brain. The colour maps represent the spatial distribution of elastic moduli in the injured and contralateral hemispheres 21 **(a)** and 23 **(b)** days PI. Four regions were identified for further quantification, including a rectangular region (dashed rectangle) around the injury site (~150 μm width centred at the scar). **(c, d)** Comparison of the elastic properties of these regions. **(e, f)** Average relative drop in elastic modulus of the regions indicated in **(a,b)** compared to the uninjured contralateral hemisphere. Error bars are SEM, *** P<0.001.

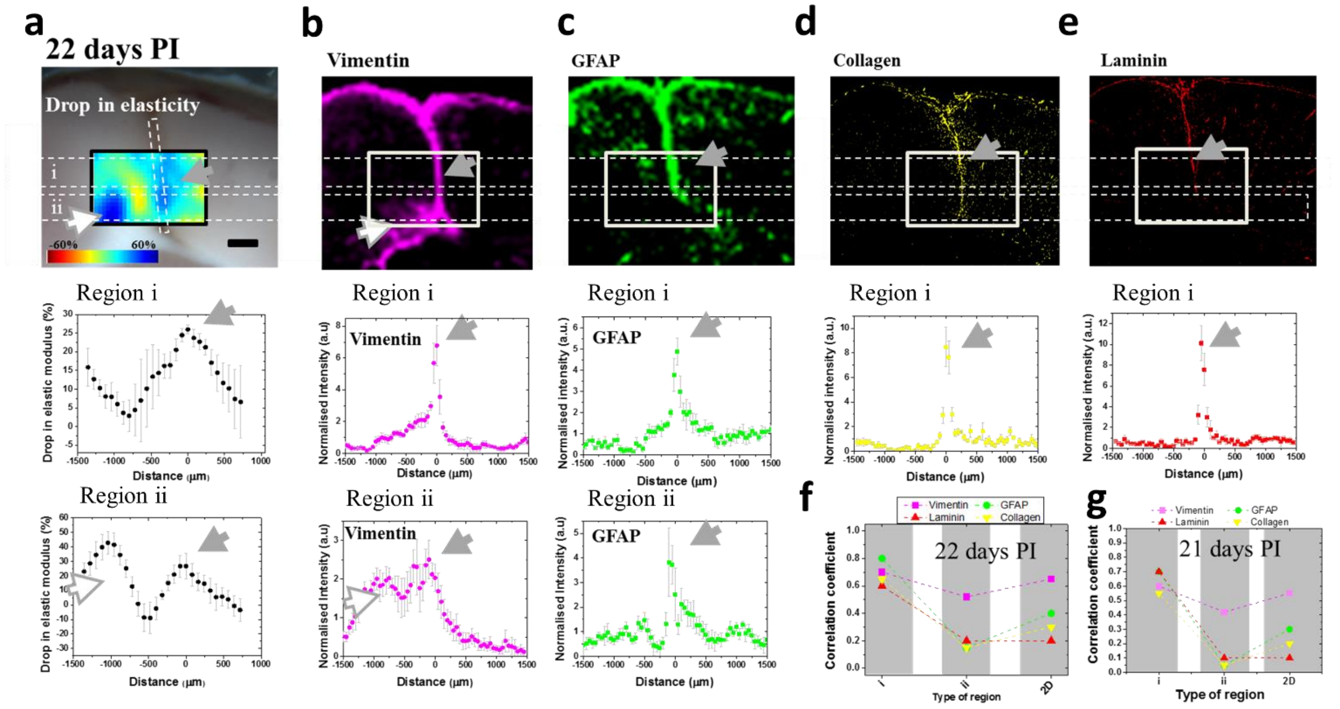

Supplementary Figure 4: **Correlation between tissue mechanics, gliosis, and ECM changes at 22 days PI.** (*cf.* Fig. 5 for an earlier time point) **a)** Rainbow-pallet map showing the relative difference in elastic modulus between injured and uninjured cortical hemisphere. Scale bar=500 µm. The panels in the bottom rows represent vertical projection profiles (mean ± SD) of the changes in elasticity determined for the regions i and ii indicated by dashed lines in the map. **(b, c)** The normalised and down-scaled pixel intensity image of vimentin and GFAP expression. The panels in the bottom rows represent vertical projection profiles (mean ± SD) of the vimentin and GFAP normalised intensity estimated for the regions i and ii indicated by dashed lines in their respective images **(d, e)** The normalised and down-scaled pixel intensity image of collagen IV and laminin expression. The panels in the bottom row represent vertical projection profiles (mean ± SD) of the collagen and laminin normalised intensity in region i of their respective fluorescence images. In **(a-e)**, grey arrows indicate the direct positive correlation between a drop in elasticity and an increase in protein expression. A positive correlation between tissue softening and vimentin and GFAP expression was also observed in regions located far away from the injury site medial to the scar (white arrows). **(f, g)** Shown are the calculated linear correlation coefficients for regions i and ii as well as the 2D correlation coefficients derived by linearly correlating the 2D matrix of the change in elasticity and the 2D maps of fluorescence intensity for each marker, for 22 (f) and 21 (g) days PI.

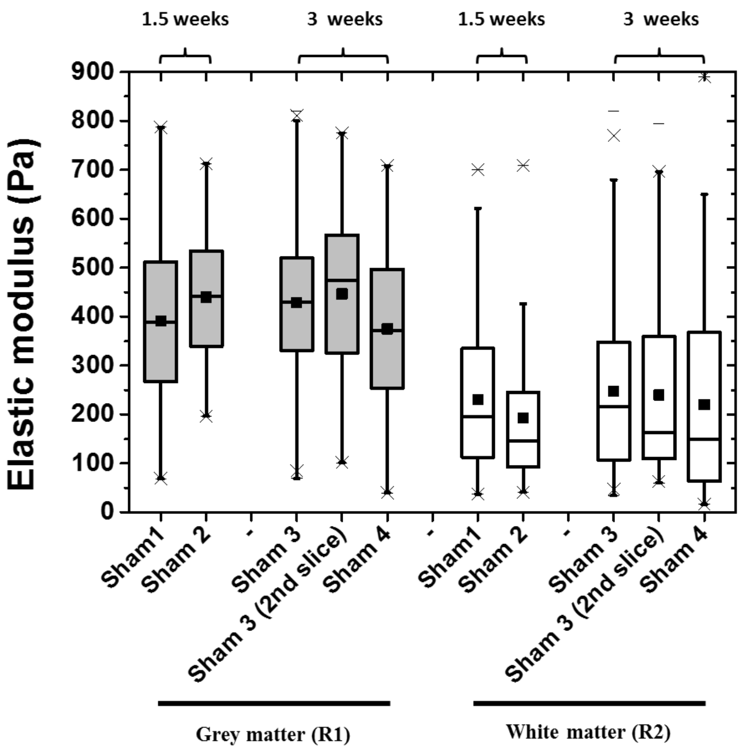

Supplementary Figure 5: Comparison of the elastic moduli of grey and white matter of control animals with sham spinal cord lesions. Inter-animal variability was low, and grey as well as white matter were statistically similar in all animals. For multi-comparison tests see **Supplementary Table 6**.

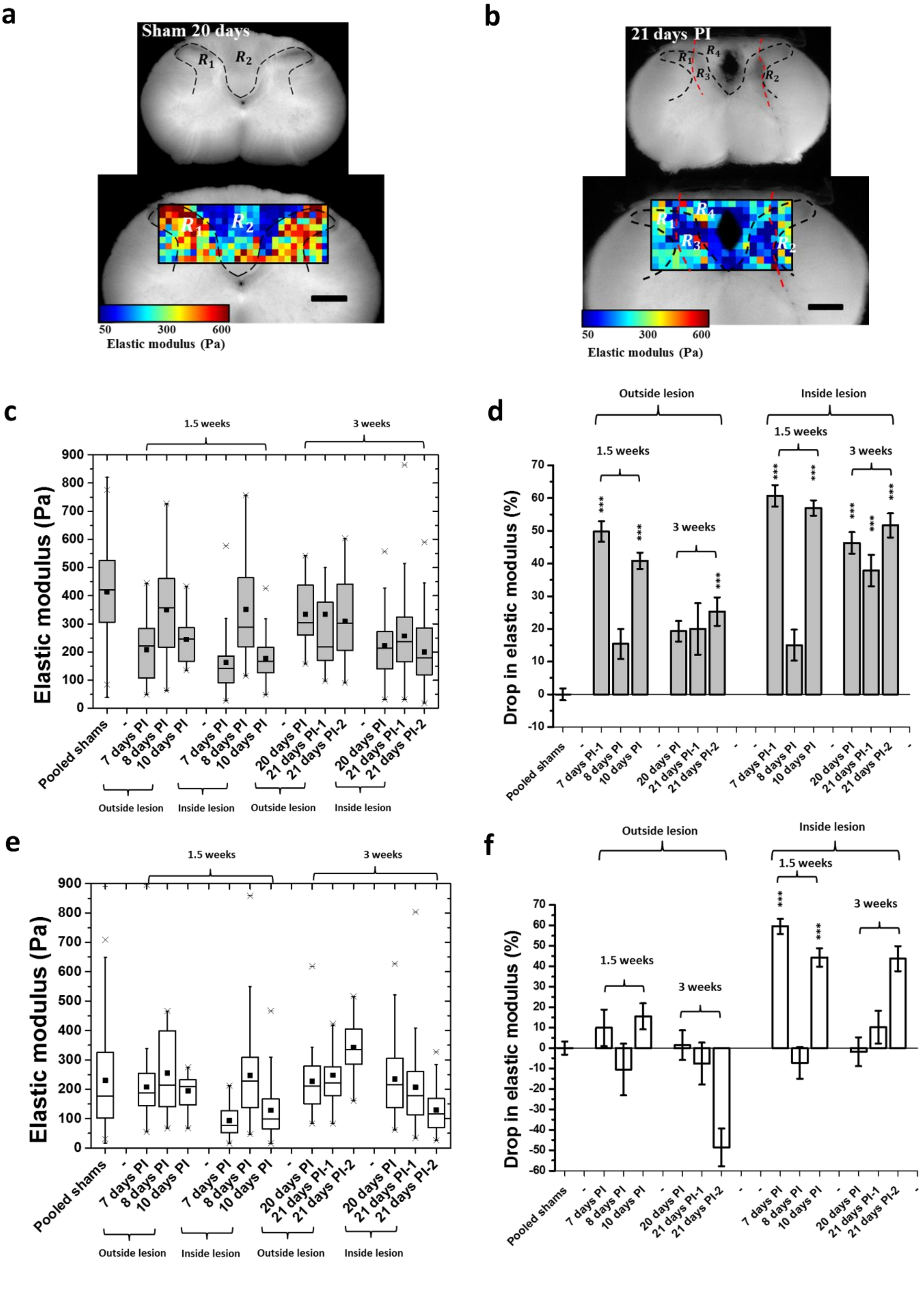

Supplementary Figure 6: Quantification of spatiotemporal changes in spinal cord tissue elasticity after crush injuries. (a) Transverse spinal cord section of a sham control at a late time point depicting the outlines of grey (R_1) and white (R_2) matter in the area of interest (dashed lines). The colour map represents the spatial distribution of elastic moduli in the tissue. **(b)** Transverse spinal cord section of an animal with a dorsal column crush lesion at 21

days PI. The approximate outlines of the injury are indicated by the red dashed lines. The colour map represents the spatial distribution of elastic moduli in both healthy and injured grey (R_1 and R_3, respectively) and healthy and injured white matter (R_2 and R_4, respectively). Scale bars=500 μm. **(c, e)** Boxplots showing the distribution of elastic moduli in the grey **(c)** and white **(e)** matter of 6 individual injured animals outside and inside the visible lesions at different time points PI in comparison to the grey and white matter of four pooled sham control animals. **(d ,f)** Average relative drop in elastic modulus of the same regions compared to the pooled sham controls. Error bars are SEM, *** $P<0.001$.

Supplementary tables

	R1'	R2	R2'	R3	R3'
**R1**	0.52	1.8E-10	6.6E-16	0.008	9.9E-04
**R1'**		1.4E-11	4.4E-17	0.009	0.01
**R2**			0.48	2.0E-16	6.6E-20
**R2'**				1.3E-23	4.6E-32
**R3**					0.75

Supplementary Table 1: Statistical comparison between the elastic moduli of different cortical regions shown in Fig. 1. Brain tissue is bilaterally symmetric; tissue regions are mechanically similar in both brain hemispheres. Highlighted in red indicates P<0.001.

	9 days	10 days	21 days	22 days	23 days
**8 days**	0.82	0.21	0.99	0.48	0.87
**9 days**		0.68	0.18	0.97	0.094
**10 days**			0.013	0.96	0.0175
**21 days**				0.026	0.88
**22 days**					0.021

Supplementary Table 2: Statistical analysis of the variability of brain tissue mechanical properties between the control maps of the different animals. All elasticity maps of uninjured brain tissue are mechanically similar.

	9 days	10 days	21 days	22 days	23 days
**8 days**	0.21	0.19	2.070E-08	6.915E-06	4.097E-08
**9 days**		0.99	2.068E-08	2.069E-08	2.068E-08
**10 days**			2.068E-08	2.068E-08	2.068E-08
**21 days**				0.44	0.93057
**22 days**					0.94

Supplementary Table 3: Statistical analysis of the variability of brain tissue stiffness at the injury site after cortical stab injuries at ~1.5 weeks and ~three weeks PI. Measurements are very reproducible: within one group, i.e., at the same time point, tissue stiffness is comparable. However, injured tissue is significantly softer at ~1.5 weeks than at ~three weeks PI. Highlighted in red indicates P<0.001.

		1.5 weeks						3 weeks						
		**Lateral**	**Ctr around**	**Around**	**Scar**	**Ctr medial**	**Medial**	**Ctr lateral**	**Lateral**	**Ctr around**	**Around**	**Scar**	**Ctr medial**	**Medial**
1.5 weeks	**Ctr lateral**	0.998	0.009	3.5E-07	3.5E-07	3.5E-07	3.5E-07	0.0586	3.5E-07	3.5E-07	3.5E-07	3.5E-07	0.011	3.5E-07
	**Lateral**		0.193	3.5E-07	3.5E-07	7.4E-07	3.5E-07	0.4831	8.4E-07	3.5E-07	3.5E-07	3.5E-07	0.242	3.5E-07
	**Ctr around**			3.6E-07	3.5E-07	0.022	3.5E-07	0.998	0.041	2.1E-04	3.5E-07	3.5E-07	0.999	3.5E-07
	**Around**				3.5E-07	0.390	0.999	4.1E-07	0.072	0.693	1.1E-06	3.5E-07	3.5E-07	0.451
	**Scar**					3.5E-07	3.5E-07	3.5E-07	3.5E-07	3.5E-07	3.5E-07	4.3E-04	3.5E-07	3.5E-07
	**Ctr medial**						0.009	0.025	0.997	0.999	3.5E-07	3.5E-07	0.006	3.4E-05
	**Medial**							3.5E-07	0.006	0.167	5.0E-04	3.5E-07	3.5E-07	0.986
3 weeks	**Ctr lateral**								0.048	0.001	3.5E-07	3.5E-07	0.987	3.5E-07
	**Lateral**									0.994	3.5E-07	3.5E-07	0.009	4.3E-07
	**Ctr around**										3.5E-07	3.5E-07	1.7E-05	6.4E-05
	**Around**											3.5E-07	3.5E-07	0.014
	**Scar**												3.5E-07	3.5E-07
	**Ctr medial**													3.5E-07

Supplementary Table 4: Statistical comparison between the elastic moduli of the different cortical regions shown in Figs. 3a and 3b. Scar tissue is significantly softer than the healthy brain. In particular, the white matter tracts-containing more distant tissue regions are softer than in control tissue. Highlighted in red indicates $P<0.001$.

		1.5 weeks			
		**GFAP**	**Vimentin**	**Laminin**	**Collagen**
3 weeks	**GFAP**	0.02			
	**Vimentin**		0.18		
	**Laminin**			0.003	
	**Collagen**				0.004

Supplementary Table 5: Statistical comparison between the levels of protein expression at different time points. While intermediate filament levels remain high until at least three weeks PI, laminin and collage IV levels significantly drop at three weeks PI after a strong rise at 1.5 weeks PI.

		Grey matter (R1)				White matter (R2)				
		Sham 2	Sham 3	Sham 3 (2nd slice)	Sham 4	Sham 1	Sham 2	Sham 3	Sham 3 (2nd slice)	Sham 3
Grey matter (R1)	Sham 1	0.742	0.790	0.398	0.999	1.3E-07	1.3E-07	1.3E-07	1.3E-07	1.3E-07
	Sham 2		0.98	0.999	0.343	1.3E-07	1.3E-07	1.3E-07	1.3E-07	1.3E-07
	Sham 3			0.999	0.312	1.3E-07	1.3E-07	1.3E-07	1.3E-07	1.3E-07
	Sham 3 (2nd slice)				0.095	1.3E-07	1.3E-07	1.3E-07	1.3E-07	1.3E-07
	Sham 4					1.6E-07	1.3E-07	2.4E-07	1.8E-07	1.3E-07
White matter (R2)	Sham 1						0.889	0.999	0.999	0.999
	Sham 2							0.362	0.638	0.986
	Sham 3								0.999	0.970
	Sham 3 (2nd slice)									0.998

Supplementary Table 6: Statistical analysis of the variability of spinal cord tissue mechanics between the control animals. Spinal cords of all four sham animals were mechanically similar. Furthermore, grey matter was significantly stiffer than white matter. Shams 1 and 2 are healthy animals tested at ~1.5 weeks PI, and shams 3 and 4 at ~three weeks PI. Highlighted in red indicates P<0.001.

		Outside lesion						Inside lesion					
		7 days PI	8 days PI	10 days PI	20 days PI	21 days PI-1	21 days PI-2	7 days PI	8 days PI	10 days PI	20 days PI	21 days PI-1	21 days PI-2
	Control	2.8E-07	0.670	4.5E-04	0.073	0.089	0.001	2.8E-07	0.083	2.8E-07	2.8E-07	2.8E-07	2.8E-07
Outside lesion	7 days PI		0.028	1.000	0.035	0.059	0.277	0.977	0.001	1.000	1.000	0.968	1.000
	8 days PI			0.570	1.000	1.000	0.998	3.6E-06	1.000	0.000	0.016	0.306	0.001
	10 days PI				0.713	0.778	0.970	0.714	0.312	0.929	1.000	1.000	0.997
	20 days PI					1.000	1.000	7.6E-07	1.000	3.7E-05	0.014	0.396	***
	21 days PI-1						1.000	3.5E-06	1.000	1.4E-04	0.032	0.524	0.001
	21 days PI-2							1.8E-04	0.983	0.004	0.240	0.933	0.024
Inside lesion	7 days PI								2.8E-07	1.000	0.469	0.018	0.937
	8 days PI									2.9E-07	0.000	0.028	0.000
	10 days PI										0.917	0.178	1.000
	20 days PI											0.987	1.000
	21 days PI-1												0.578

Supplementary Table 7: Statistical comparison between the elastic moduli of grey matter within regions inside and outside of the injury for 6 animals. Spinal cord grey matter softens after crush injuries. . Highlighted in red indicates P<0.001.

		Outside lesion						Inside lesion					
		7 days PI	8 days PI	10 days PI	20 days PI	21 days PI-1	21 days PI-2	7 days PI	8 days PI	10 days PI	20 days PI	21 days PI-1	21 days PI-2
Outside lesion	Control	0.999	1.000	0.999	1.000	1.000	0.004	4.0E-07	1.000	1.2E-06	1.000	0.991	0.022
	7 days PI		0.999	1.000	1.000	0.999	0.008	0.015	0.976	0.181	0.999	1.000	0.590
	8 days PI			0.999	1.000	1.000	0.908	0.067	1.000	0.296	1.000	0.999	0.471
	10 days PI				1.000	0.998	0.080	0.510	0.990	0.932	0.999	1.000	0.977
	20 days PI					1.000	0.057	0.001	1.000	0.025	1.000	1.000	0.230
	21 days PI-1						0.629	0.010	1.000	0.091	1.000	0.998	0.267
	21 days PI-2							2.8E-07	0.142	2.9E-07	0.059	0.002	2.9E-06
Inside lesion	7 days PI								0.000	0.988	8.0E-05	0.004	0.998
	8 days PI									7.9E-05	1.000	0.931	0.020
	10 days PI										0.002	0.070	1.000
	20 days PI											0.998	0.085
	21 days PI-1												0.480

Supplementary Table 8: Statistical comparison between the elastic moduli of white matter within regions inside and outside of the injury for 6 animals in spinal cord experiments. Spinal cord white matter is less prone to mechanical changes after crush injury, although it softens significantly at the injury site in ~50% of the cases. *** P<0.001.
